# Evaluation of low acuity patients discharged from a virtual emergency department at a major urban academic health sciences centre in Toronto, Canada.

**DOI:** 10.23889/ijpds.v7i3.1926

**Published:** 2022-08-25

**Authors:** Emily Borgundvaag, Lesley Plumptre, Michael Paterson, Diana An, Shelley McLeod, Jean-Eric Tarride, Clare Atzema, Michael Schull, Aikta Verma, Justin Hall

**Affiliations:** 1 Institute for Clinical Evaluative Sciences (ICES); 2 Schwartz/Reisman Emergency Medicine Institute (SREMI), Sinai Health; 3 Department of Health Research Methods, Evidence and Impact (HEI), McMaster University; 4 University of York

## Objectives

In response to the COVID-19 pandemic, Sunnybrook Health Sciences Centre launched the first virtual emergency department (VED) in Toronto, Ontario. The objective of this pilot project was to leverage linked administrative data to describe the healthcare utilization of VED patients compared to matched patients who attended an ED in person.

## Approach

Evaluation of the VED program was supported by the ICES Applied Health Research Question Program, which is funded by the Ontario Ministry of Health to answer questions directly related to Ontario healthcare policy, planning, or practice. VED visit records from December 2020 to May 2021 were linked with Ontario administrative data. VED patients with low acuity complaints were matched 1:1 with in-person ED comparators according to visit date, presenting complaint, and a propensity score that incorporated age, sex, comorbidities, and other important potential confounders. The primary outcomes were healthcare utilization within 7 days and all-cause mortality within 30 days.

## Results

Of the 609 eligible patients discharged from the VED, 600 (98.5%) were successfully matched to a comparator. Mean (SD) age was 43.0 (21.1) and 64.1% were female. In-person ED revisits and hospitalizations were similar for VED and comparator patients at 72 hours (ED: 12.1% vs. 11.3%; Δ 0.8%, 95%: -2.8, 4.5%; hospitalization: 1.2% vs. 1.5%; Δ 0.3%, 95%: -0.7, 1.4%,) and 7 days (ED: 16.1% vs. 14.4%; Δ 1.7, 95%: -2.4, 5.7%; hospitalization: 1.7% vs. 1.8%; Δ 0.2%, 95%: -0.1, 1.4%) following the index visit. The number of patients visiting a primary care provider within 7 days was also similar between groups (36.7% vs. 32.4%; Δ 4.3, 95%: -1.1, 9.8%). No patients died within 30 days.

## Conclusion

VED patients and their matched comparators had similar healthcare utilization in the 7 days following their index ED visit. Methodology from this study will inform a province-wide evaluation of VED programs across Ontario.

